# Editorial: Preclinical macaque models of viral diseases

**DOI:** 10.3389/fimmu.2023.1331774

**Published:** 2023-11-09

**Authors:** Jeremy Smedley

**Affiliations:** Vaccine and Gene Therapy Institute, Oregon Health & Science University, Beaverton, OR, United States

**Keywords:** macaque, model, HIV, SIV, transplantation, Zika (ZIKV), viral disease

Preclinical macaque models of viral diseases are among the most translatable animal models to human patients (1–3). These models have key benefits including rigorously controlled timing of manipulations, routes and doses of infectious agents and interventions, age and health status of animals, etc., which can serve to eliminate important sources of variability and potential confounding variables. Macaques have significant genetic and physiological similarities to humans and their immune responses to pathogens and vaccines are highly relevant and, where appropriate models are employed, highly translatable (4–10). An essential element of macaque experiments is the ability to sample tissues in ways and at frequencies that are simply not possible in human patients. This can be done using minimally invasive sampling techniques that provide high quality samples with optimal animal welfare and minimal complications (11, 12). Serial sampling of mesenteric lymph nodes (MLN), spleen, and liver have been performed laparoscopically and combined with sampling of peripheral lymph nodes, bone marrow, bronchoalveolar lavage, mucosal samples (GI, vaginal, oral), CSF and blood during a single anesthetic event providing comprehensive information from key sites throughout the body. These samples allow for assessment of immunity, inflammation, and host-pathogen interactions at critical time points such as baseline, pre/post vaccine or intervention, and/or pre/post challenge (13–18). This sampling can be combined with imaging for targeted sampling to ensure that localized events, such as interactions at local draining lymph nodes, are appropriately captured (19). This sampling can be done even in the face of significant immunosuppression, such as is necessary to perform hematopoietic stem cell transplant and modeling HIV reservoir elimination/functional cure similar to what has occurred in a handful of human patients. The results of this model have provided critical insights demonstrating that the last place intact Simian Immunodeficiency Virus (SIV) can be detected in macaques that go on to be functionally cured is the MLN. The model further demonstrated that once intact SIV is no longer detected at this site the animals remain aviremic upon antiretroviral therapy (ART) cessation; an important finding that can help guide physicians attempting to achieve the best outcomes for their patients (14). However, the need for indwelling catheters and multiple surgeries combined with immunosuppression for successful allogeneic transplantation can promote the risk of infections that can impact animal welfare and present a risk to occupationally exposed staff. Therefore, assessment of the health status of the animals and ensuring that their background pathogen status going into these critical experiments is optimized for the health of the macaques as well as the safety of occupationally exposed staff is essential. One of the accepted manuscripts evaluated the baseline status of the cynomolgus macaques prior to project use and found high levels of not just Methicillin resistant *Staphylococcus aureus* (MRSA), but Vancomycin resistance (VRSA) as well. They then demonstrated successful decolonization and long-term maintenance of animals free of these critical organisms, improving the health/welfare of the animals and staff safety (Bochart et al.).

Two additional manuscripts also evaluated transplant models related to prevention/elimination of HIV in relevant macaque models. These manuscripts evaluated autologous transplantation of two different Chimeric Antigen Receptor (CAR) T cells aimed at preventing or eliminating SIV or Simian-Human Immunodeficiency Virus (SHIV) in macaque models of acquisition and/or ART suppressed infection. The first article looked at the use of a conserved elements vaccine and with exvivo expansion and autologous transplantation to increase the number of polyfunctional SHIV specific T cells present at the time of SHIV challenge (Dross et al.). They demonstrated the safety of this approach and, whereas they were unable to demonstrate significant efficacy, the results mirrored those seen in the clinic demonstrating that the macaque model offers a highly translatable method to evaluate the immunology, safety and virological outcomes for CAR T cell therapies prior to use in patients. The second article evaluated the use of a CD20 depleting antibody prior to administration of SIV-specific CD4-MBL-CAR T cells expressing the follicular homing receptor CXCR5, resulting in increased follicular homing of the CAR T cells (Pampusch et al.). Sampling of key lymphoid sites demonstrated reduced efficacy of the CD20 depletion in lymph nodes compared to blood as well as the increased homing of the CAR T cells to this location in CD20 depleted animals. However, despite the increased number of CAR T cells the animals failed to control the SIVmac239 infection upon ART withdrawal and the depleted animals demonstrated Cytokine Release Syndrome (CRS) with elevated levels of IL-6 that required treatment with anti-IL-6 antibodies to prevent mortality; an important safety consideration for potential translation of these therapies to the clinic.

Another accepted article evaluated similarities and differences in the fragment crystallizable (Fc) mediated functions of antibodies between rhesus macaques and humans (Tolbert et al.). Looking at the FcγIII functions, which play a significant role in antibody mediated effector function, they demonstrated important differences between humans and macaques that further our understanding of how to interpret results of macaque studies used to evaluate vaccines and antibody-based therapeutics that involve or depend on Fc-effector functions. The final article looked at a macaque model of Zika virus infection (Krabbe et al.). Pregnant macaques were inoculated during the first trimester of pregnancy and half demonstrated clearance of the virus from the maternal circulation by 7 days post infection (DPI). The other half took longer to eliminate viral RNA from the maternal plasma and had higher levels of vRNA at the maternal-fetal interface at the time of delivery. Outcomes were determined in the first 7 DPI with higher antibody titers associated with worse outcomes indicating that antibodies were not associated with maternal control and could be a potential biomarker for worse outcomes.

These articles demonstrate the potential macaques represent as powerful translatable infectious disease models permitting interrogation of key questions involving pathogen-host interactions as well as evaluating the efficacy of vaccines, therapeutics and other interventions and how further improvements in the models and the background health of the animals will allow for improved sampling, improved animal care and welfare, improved human health and safety, and elimination of critical sources of variability leading to even more rigorous and reproducible experiments.

## Author contributions

JS: Writing – original draft.
